# The causality between rheumatoid arthritis and postural deformities: bidirectional Mendelian randomization study and mediation analysis

**DOI:** 10.3389/fimmu.2024.1453685

**Published:** 2024-10-03

**Authors:** Piqian Zhao, Zhe Chen, Ya Wen, Hongtao Zhang, Liangyuan Wen, Zijie Pei

**Affiliations:** ^1^ Beijing Hospital, National Center of Gerontology, Institute of Geriatric Medicine, Beijing, China; ^2^ Chinese Academy of Medical Sciences & Peking Union Medical College, Beijing, China; ^3^ Department of Orthopedics, The First Affiliated Hospital of Soochow University, Soochow University, Suzhou, Jiangsu, China; ^4^ Department of Thoracic Surgery, The Fourth Affiliated Hospital of Soochow University, Suzhou, China; ^5^ Capital Medical University School of Biomedical Engineering, Beijing, China

**Keywords:** rheumatoid arthritis, postural deformities, Mendelian randomization, hallux valgus, flat foot, scoliosis

## Abstract

**Background:**

To better understand the preventive or therapeutic clinical interventions that may be supported by the association between rheumatoid arthritis (RA) and postural deformities including hallux valgus, flat foot, and scoliosis, this study was conducted using Mendelian randomization (MR) analysis. It aimed to investigate whether RA is causally associated with postural deformities in European populations.

**Methods:**

Summary-level data on RA and postural deformities were obtained from the IEU OpenGWAS project and Finngen database, respectively. LDSC regression analysis was conducted to assess the genetic correlation between these diseases. The inverse variance weighting (IVW) method was employed as the primary approach for two-sample MR analyses to evaluate causality. Supplementary methods included MR-Egger, maximum likelihood, weighted median, and cML-MA. To test for potential horizontal pleiotropy, we performed the MR-Egger intercept test, cML-MA, and secondary analyses after excluding confounders. Additionally, mediation analyses were conducted using two-step MR.

**Results:**

The IVW method revealed RA to be causally associated with hallux valgus (OR 1.132, 95% CI 1.087-1.178, P < 0.001) and flat foot (OR 1.197, 95% CI 1.110-1.291, *P* < 0.001). Among postural deformities, hallux valgus was causally associated with flat foot (OR 1.823, 95% CI 1.569-2.119, *P* < 0.001) and scoliosis (OR 1.150, 95% CI 1.027-1.287, *P* < 0.05). No significant horizontal pleiotropy was detected. Moreover, mediation analyses indicated that hallux valgus mediates the effect of RA on flat foot (mediation effect 0.024, 95% CI 0.005-0.044, *P* < 0.05), with a mediation proportion of 41.31%.

**Conclusion:**

These findings indicate a potential causal association between genetically predicted RA and both hallux valgus and flat foot. Furthermore, hallux valgus serves as a mediator in the pathway from RA to flat foot. This underscores the importance of early screening and preventive treatment of foot deformities in RA patients. Further research is necessary to determine the applicability of these findings in non-European populations.

## 1 Introduction

Rheumatoid arthritis (RA) is a systemic autoimmune disease that affects the small joints and causes chronic synovitis with progressive development, ultimately leading to joint destruction and deformity (1). The prevalence of RA in Europe and North America is 0.5-1.0%, and women are two to three times more affected than men (2). Genetic factors account for approximately 50-60% of the risk of developing RA, and genome-wide association studies (GWAS) using single nucleotide polymorphisms (SNPs) have identified more than one hundred loci associated with the risk of rheumatoid arthritis (3). A systemic inflammatory response in articular cartilage and bone is the main characteristic of this disease (1). Currently, pharmacological treatments such as glucocorticoids and disease-modifying antirheumatic drugs (DMARDs) are recognized for their efficacy in alleviating symptoms and decelerating the progression of RA (4). However, prolonged use of these medications is associated with serious adverse effects, including infections and liver damage. Furthermore, research in traditional Chinese medicine has demonstrated that extracts and formulations derived from various snow lotus, tea, and ginseng exhibit significant therapeutic efficacy and safety potential in the treatment of RA (5–7). In recent years, RA has been reported to have a causal relationship with orthopedic diseases, including osteoporosis, fractures, and osteochondral malignant neoplasm (8, 9). However, it is not clear whether RA causes the development of postural deformities.

Common postural deformities are malformations of any bodily part or component that result in postural abnormalities, which mainly include spinal deformities (scoliosis, kyphosis, and lordosis), knee deformities (knock knees and bow legs), and foot deformities (hallux valgus and flat foot) (10–13). Previous observational studies have revealed that hallux valgus, flat foot and scoliosis are each positively correlated with RA. A comprehensive review reported studies on the correlation between RA and foot problems (two-thirds of the studies were from European populations). They found that 35.0-65.3% and 11.0-42.1% of RA patients suffered from hallux valgus and flat foot, respectively, and hallux valgus was considered to be the most common foot deformity in RA patients. Hallux valgus is characterized by the medial deviation of the first metatarsal as well as lateral hallux deviation, often leading to foot pain and even dyskinesia (14). In RA, forefoot deformities may be associated with flat foot. A positive correlation between hallux valgus and flat foot has been reported, demonstrating that the calcaneal pitch angle and lateral talocalcaneal angle were significantly lower in the hallux valgus group than in controls (15). The prevalence of flat foot in adults ranges from 5.0-14.0%, and the foot is manifested by decreased medial longitudinal arch and valgus hindfoot (16). Arch collapse can affect a patient’s gait and motor function. The etiology of hallux valgus and flat foot remains unclear and is currently thought to be primarily related to genetic factors, footwear habits, neuromuscular disorders, and inflammatory disorders (13). RA and scoliosis correlation has been widely investigated in East Asian populations (17, 18). A Chinese case–control study reported that 42.6% of RA patients had scoliosis, significantly higher than controls (19). To our knowledge, there are no reported studies on the association of RA with scoliosis in European populations, which needs to be explored. Although more established surgical procedures are available for the correction of postural deformities including hallux valgus, flat foot, and scoliosis, the high expense of the surgery and relatively low postoperative satisfaction aggravate the burden on the patients (20–22). It is crucial to actively explore the etiology and prevention strategies for postural deformities.

**Figure 1:**
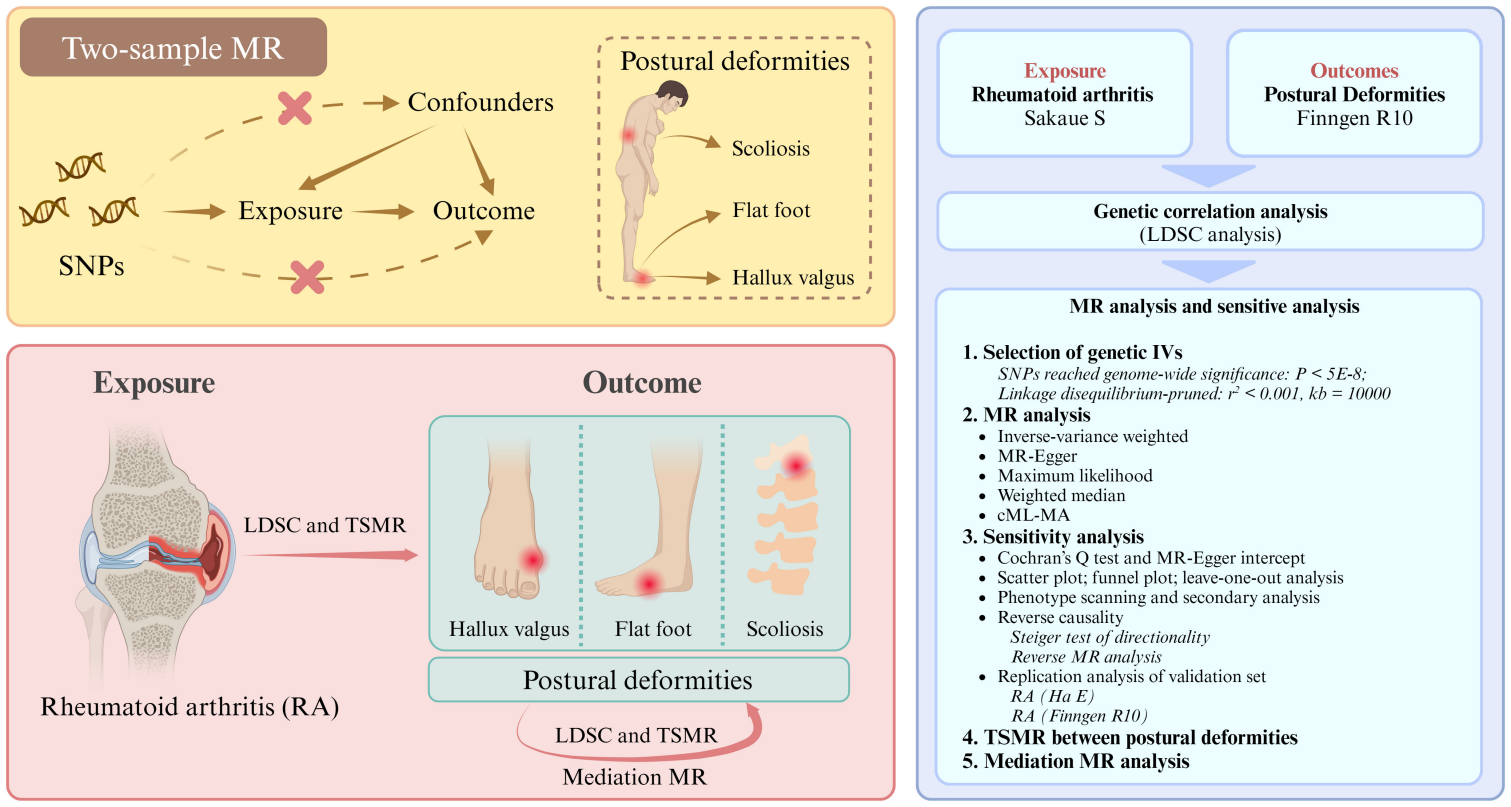
Research flowchart. SNPs, single nucleotide polymorphisms. LDSC, linkage disequilibrium score; TSMR, two-sample Mendelian randomization.

Observational studies cannot provide support for causality due to the inability to control for potential confounders and reverse causality (23). Typically, randomized controlled trials (RCT) are the gold standard for causal inference in epidemiological studies. However, because of ethical issues, RCT may not be applied to study the causal relationship between RA and postural deformities (24). Mendelian randomization (MR) is another epidemiological method for inferring causality, and it uses SNPs as instrumental variables (IVs) to evaluate causal associations between exposure factors and outcome events (25, 26). As genetic variants are randomly assigned at the formation of fertilized eggs, they are not affected by external factors or disease states. Thus, MR studies can compensate for the limitations of observational studies and achieve the same effects as RCT, greatly reducing the expense of the study (27). To our knowledge, there are currently no MR studies on the association between RA and postural deformities. This study aims to utilize MR to investigate the potential causal associations between RA and three postural deformities— hallux valgus, flat foot, and scoliosis— as well as the interrelationships among these deformities, in a European population (**Figure 1**).

## 2 Methods

This study is reported according to the STROBE-MR statement (**Supplementary Table S1**) (28). All data were obtained from public databases. The relevant studies were approved by the appropriate ethical review board (29, 30).

### 2.1 Study design and data sources

The flowchart of our study design is shown in **Figure 1** (Built by the Biorender). Details of the GWAS data sources are shown in **Table 1**. The relevant GWAS datasets were all obtained from the IEU OpenGWAS project (https://gwas.mrcieu.ac.uk/) and the Finngen R10 database (https://r10.finngen.fi/). The RA dataset used for the main analysis consisted of 8,255 cases and 409,001 controls (ebi-a-GCST90018910, Sakaue S), while the ebi-a-GCST90013534 (Ha E) and M13-RHEUMA (Finngen) datasets were used for replication analyses. The datasets for the three postural deformities, including hallux valgus (15,886 cases and 262,844 controls), flat foot (2,996 cases and 262,844 controls), and scoliosis (2,817 cases and 294,770 controls), were obtained from the Finngen R10 database. All participants were from European populations.

**Table 1:** Description of GWAS data sources and details.

Traits	Gwas ID	Author	Years	Population	Sample size (cases/controls)	PMID
Rheumatoid arthritis	ebi-a-GCST90018910	Sakaue S	2021	European	417256 (8255/409001)	34594039
Rheumatoid arthritis	ebi-a-GCST90013534	Ha E	2020	European	58284 (14361/43923)	33310728
Rheumatoid arthritis	M13-RHEUMA	Finngen	2023	European	276465 (13621/262844)	—
Flat foot	M13-FLATFOOT	Finngen	2023	European	265840 (2996/262844)	—
Hallux valgus	M13-HALLUXVALGUS	Finngen	2023	European	278730 (15886/262844)	—
Scoliosis	M13-SCOLIOSIS	Finngen	2023	European	297587 (2817/294770)	—

### 2.2 IVs selection

SNPs that reached genome-wide significance of *P* < 5×10^-8^ were selected as IVs in the RA and hallux valgus data, whereas flat foot and scoliosis set the threshold to *P* < 5×10^-7^ to obtain sufficient IVs. The genetic distance was set to 10,000 kb and r^2^ < 0.001 to eliminate the linkage disequilibrium bias. The minor allele frequency > 0.01 and the palindromic SNPs were removed. Finally, the F-statistic was calculated according to the formula: *F* = R2(N-2)/(1-R2), and strong IVs with *F* > 10 were retained for MR analysis.

### 2.3 Statistical analysis

We first performed linkage disequilibrium score (LDSC) regression analysis to evaluate the genetic correlation between RA and postural deformities. The inverse variance weighting (IVW) method was used as the main analytical method for the two-sample MR (TSMR) (31). Additionally, MR-Egger, maximum likelihood, weighted median, and cML-MA were used as supplements. Forest plots were drawn based on the results.

For sensitivity analyses, Cochran’s Q test was used as a heterogeneity test (*P* < 0.05). The MR-Egger intercept test was used to evaluate potential pleiotropy (*P* < 0.05). The IVW method was considered most reliable when horizontal pleiotropy was not observed for IVs. To further avoid the bias of the results caused by potential pleiotropic variants, we applied a method based on constrained maximum likelihood and model averaging, called cML-MA, which has a robust performance for the control of genetic pleiotropy (32). Considering that obesity phenotypes may be a confounder of foot deformities, secondary MR analysis after obesity-associated SNP exclusion was performed (33). SNP for obesity phenotypes (body mass index, body fat percentage, and weight) were searched through the PhenoScanner website (http://www.phenoscanner.medschl.cam.ac.uk/). Scatter plots, funnel plots, and leave-one-out plots were constructed to assess the impact of each SNP on the outcome. Additionally, reverse MR analysis and Steiger test were performed to determine the direction of causality.

Two-step MR was utilized to investigate potential mediating pathways between exposure and outcome. The mediation effect was obtained by multiplying the effect of exposure on the mediator with the effect of the mediator on the outcome. The Sobel test was used to assess the significance of the mediation effect (*P* < 0.05). The mediation effect was divided by the total effect to obtain the proportion of the mediation effect. Since hallux valgus, flat foot, and scoliosis were all derived from the Finngen database, there may be a sample overlap. The intercept of the genetic covariance is usually used to evaluate the rate of sample overlap. If the intercept value is not close to zero, it indicates a high sample overlap (34). Samples with high overlap were further analyzed using the MRlap method (35). All MR and statistical analyses were performed using R software (version 4.4.0, https://www.r-project.org/).

## 3 Results

### 3.1 Causal effects of RA on postural deformities

LDSC regression analysis results showed that RA was significantly correlated with flat foot (r_g_= 0.419, *P* < 0.001) and hallux valgus (r_g_ = 0.262, *P* < 0.001) (**Supplementary Figure 1A**; **Supplementary Table 3**). In contrast, there was no significant correlation between RA and scoliosis (r_g_ = 0.111, *P* = 0.288).

Based on the filtering criteria, 26 SNPs were selected as IVs for RA in the main analysis dataset (Sakaue S). The Ha E and Finngen datasets in the replication analyses were filtered with 85 and 27 SNPs for RA, respectively (**Supplementary Table S2**). Among the postural deformities, 6, 37, and 3 SNPs were selected as IVs for flat foot, hallux valgus, and scoliosis, respectively.

**Figure 2:**
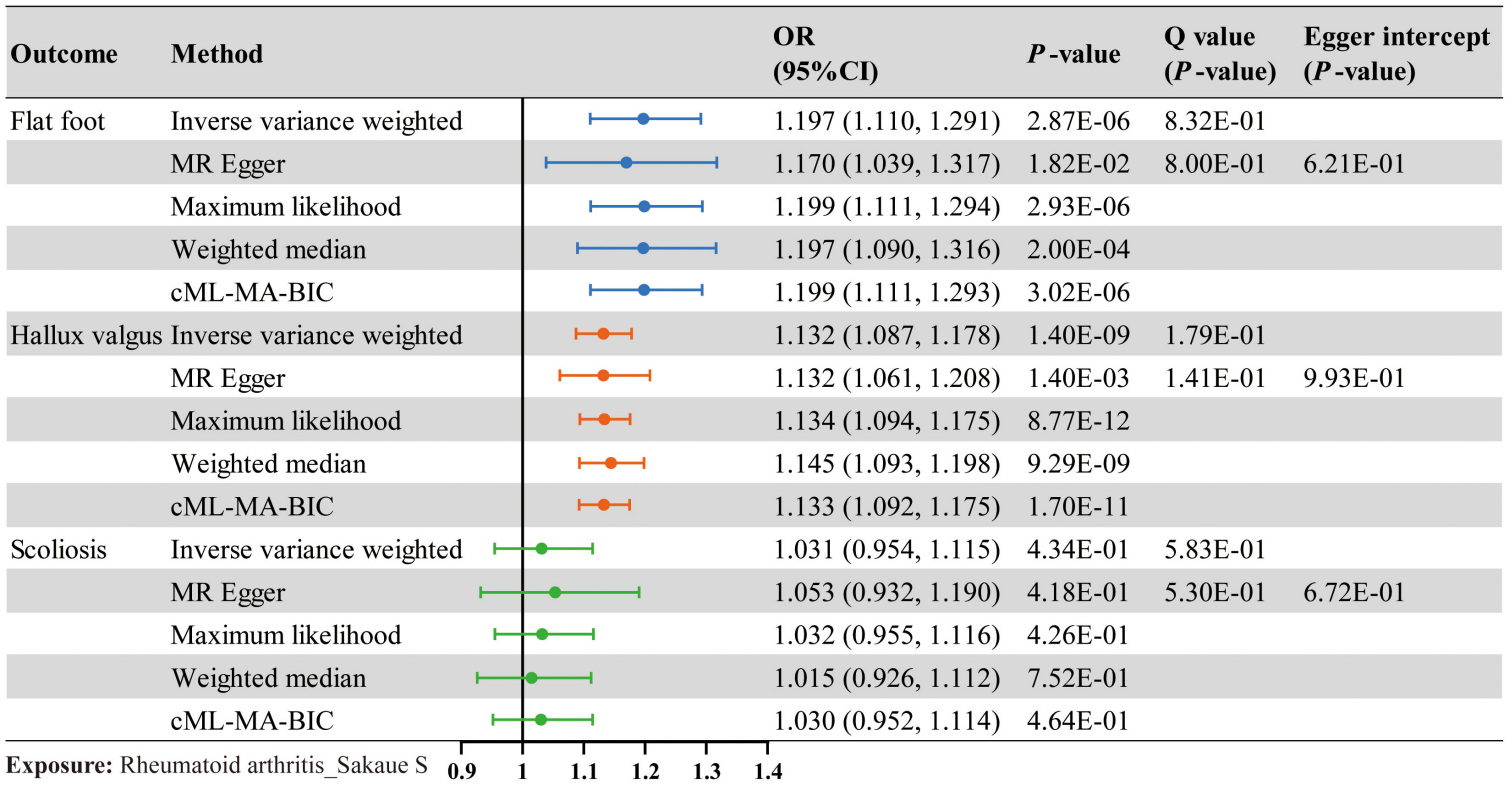
The causal effect of RA on postural deformities. Odds ratio (OR), 95% CI, and *P*-value are presented. Significance levels for heterogeneity (Cochran’s Q test) test and pleiotropy test (MR Egger intercept test) are also shown in the figure.

TSMR was performed for RA and postural deformities. The IVW method revealed a positive causality between RA and both flat foot and hallux valgus, with ORs of 1.197 (95% CI 1.110-1.291, *P* < 0.001) and 1.132 (95% CI 1.087-1.178, *P* < 0.001), respectively (**Figure 2**). Moreover, this conclusion was also supported by the MR-Egger, maximum likelihood, weighted median, and cML-MA methods (**Figures 2** **,** **3**). These five methods did not show statistical significance in evaluating the causal relationship between RA and scoliosis.

**Figure 3:**
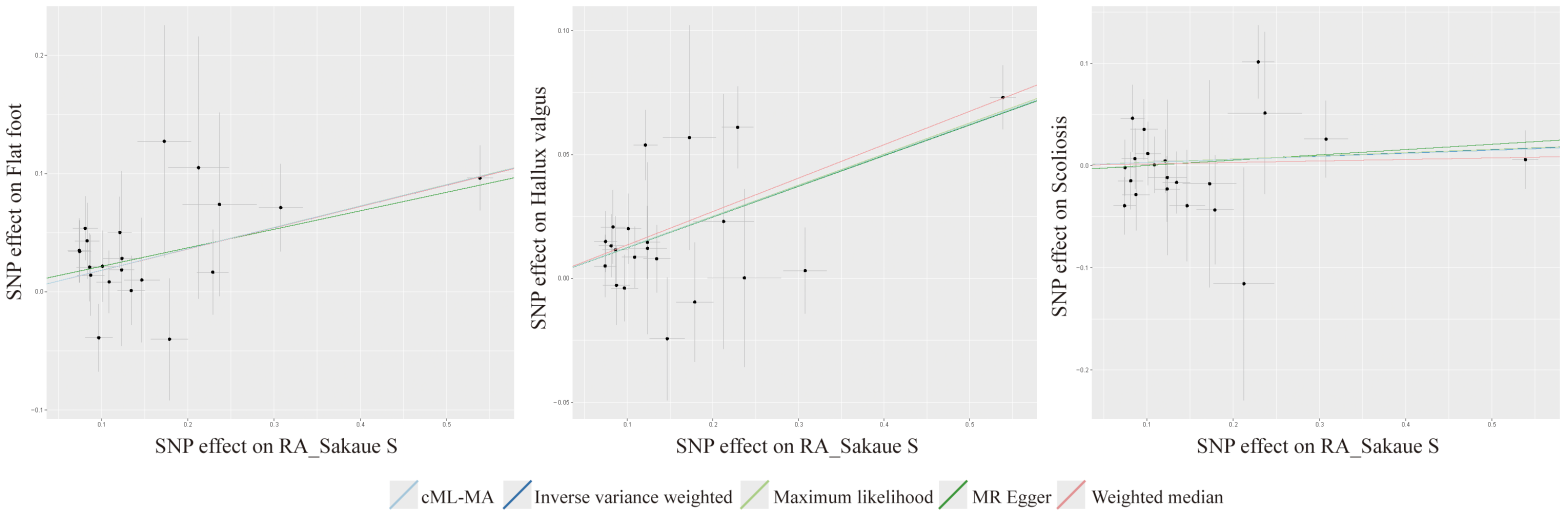
Scatter plot for MR of the causality between RA and postural deformities.

The Cochran’s Q test indicated no significant heterogeneity in the IVs included when RA was used as an exposure (**Figure 2**). And no horizontal pleiotropy was detected by the MR Egger intercept test. The results of the cML-MA method are consistent with the other four methods, indicating the findings remain reliable after considering potential horizontal pleiotropy (**Figure 2**). Scatter plots, funnel plots, and leave-one-out plots all showed the robustness of the results (**Supplementary Figures 2–4**).

Phenotypic scanning results showed that IVs in RA were associated with obesity phenotypes such as body mass index, body fat percentage, and weight, with rs35139284, rs11513729, rs58667488, rs9332735, rs6906714, rs11513729, rs2069235, and rs42034 having significant associations. After removing these SNPs, which may cause horizontal pleiotropy, the results of the secondary analysis remained consistent with the pre-removal results (**Supplementary Table S5**).

The Steiger test of directionality showed a correct positive causal relationship between RA and postural deformities. Moreover, for further validation, reverse causality was performed, and all five MR methods showed no statistically significant reverse causality (**Table 2**; **Supplementary Table S7**). Replication analyses confirmed causal associations consistent with the main analysis in two other European populations (**Supplementary Table S4**).

**Table 2:** Reverse MR analyses of RA with postural deformities.

Exposure	Outcome	Method	OR (95%CI)	*P*-value
Flat foot	Rheumatoid arthritis_Sakaue S	IVW	1.055 (0.938-1.186)	3.75E-01
Hallux valgus	Rheumatoid arthritis_Sakaue S	IVW	1.158 (0.918-1.459)	2.15E-01
Scoliosis	Rheumatoid arthritis_Sakaue S	IVW	0.998 (0.860-1.158)	9.77E-01

### 3.2 Causal effects between postural deformities

LDSC regression analysis showed significant genetic correlations between all postural deformities (**Supplementary Figure 1B**). TSMR was performed for the three postural deformities. The IVW method found a positive causal relationship between hallux valgus and flat foot (OR 1.823, 95% CI 1.569-2.119, *P* < 0.001) and scoliosis (OR 1.150, 95% CI 1.027-1.287, *P* < 0.05) (**Figure 3**; **Supplementary Table 7**). The intercept of the genetic covariance (cross trait intercept) between hallux valgus and flat foot was 0.1896 ± 0.0071, suggesting there may be a sample overlap rate, so we further analyzed by MRlap method, and the results remained consistent with the previous findings (OR 1.351, 95% CI 1.237-1.474, *P* < 0.001) (**Figure 3**; **Supplementary Table S8**). Although the IVW and MRlap methods showed that there was also a significant causal relationship between the flat foot and hallux valgus, the Steiger test suggested misdirection (*P* = 0.329, FALSE), suggesting no reverse causality between hallux valgus and flat foot (**Figure 3**). Additionally, the IVW method showed a significant causality between scoliosis and hallux valgus, but lost significance after controlling for pleiotropy with the cML-MA method (**Figure 3**).

### 3.3 Mediation analysis

**Figure 4:**
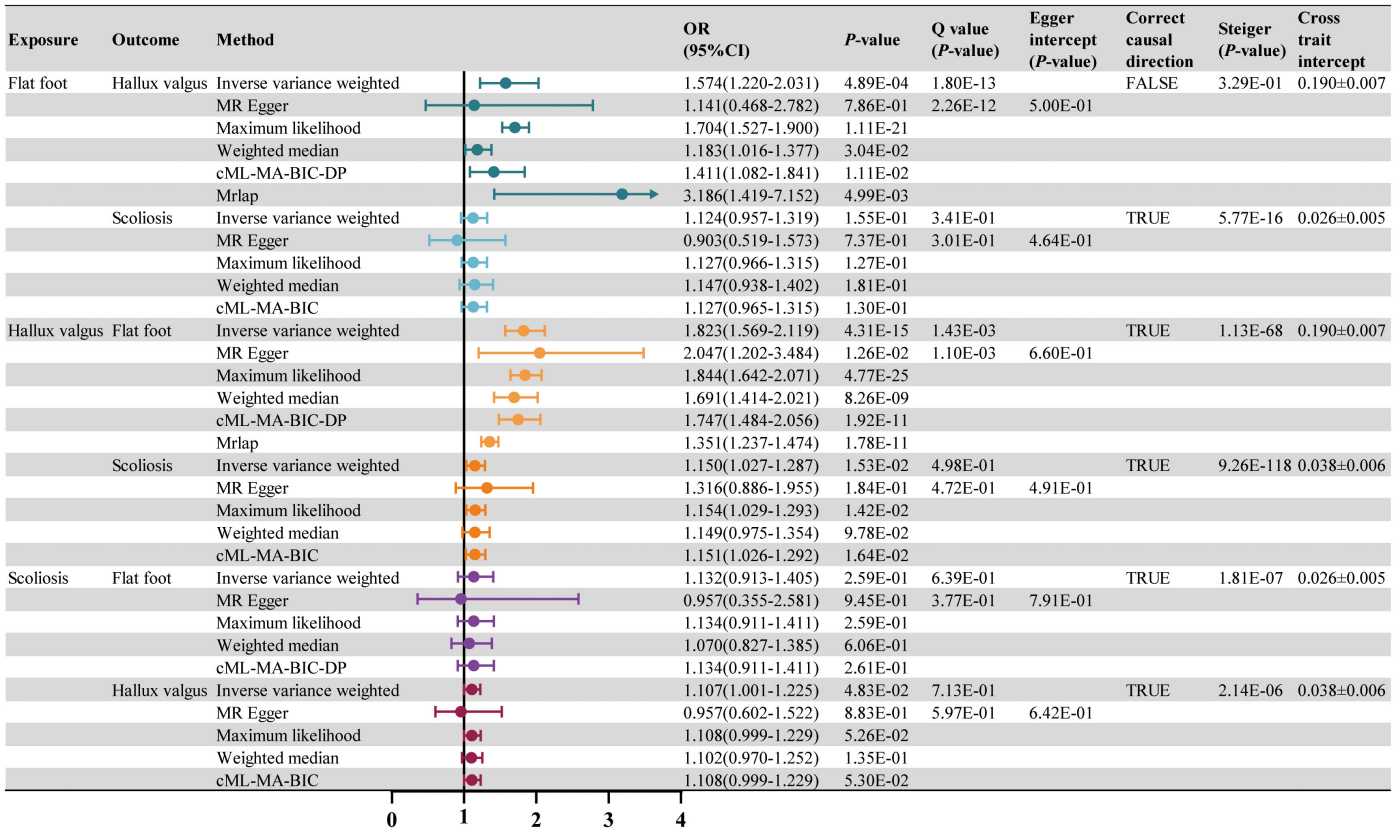
The causal effect of RA on postural deformities. OR, 95% CI and *P*-value are presented. Significance levels for Cochran’s Q test, MR Egger intercept test, Steiger test and the cross trait intercept are also shown in the figure.

**Figure 5:**
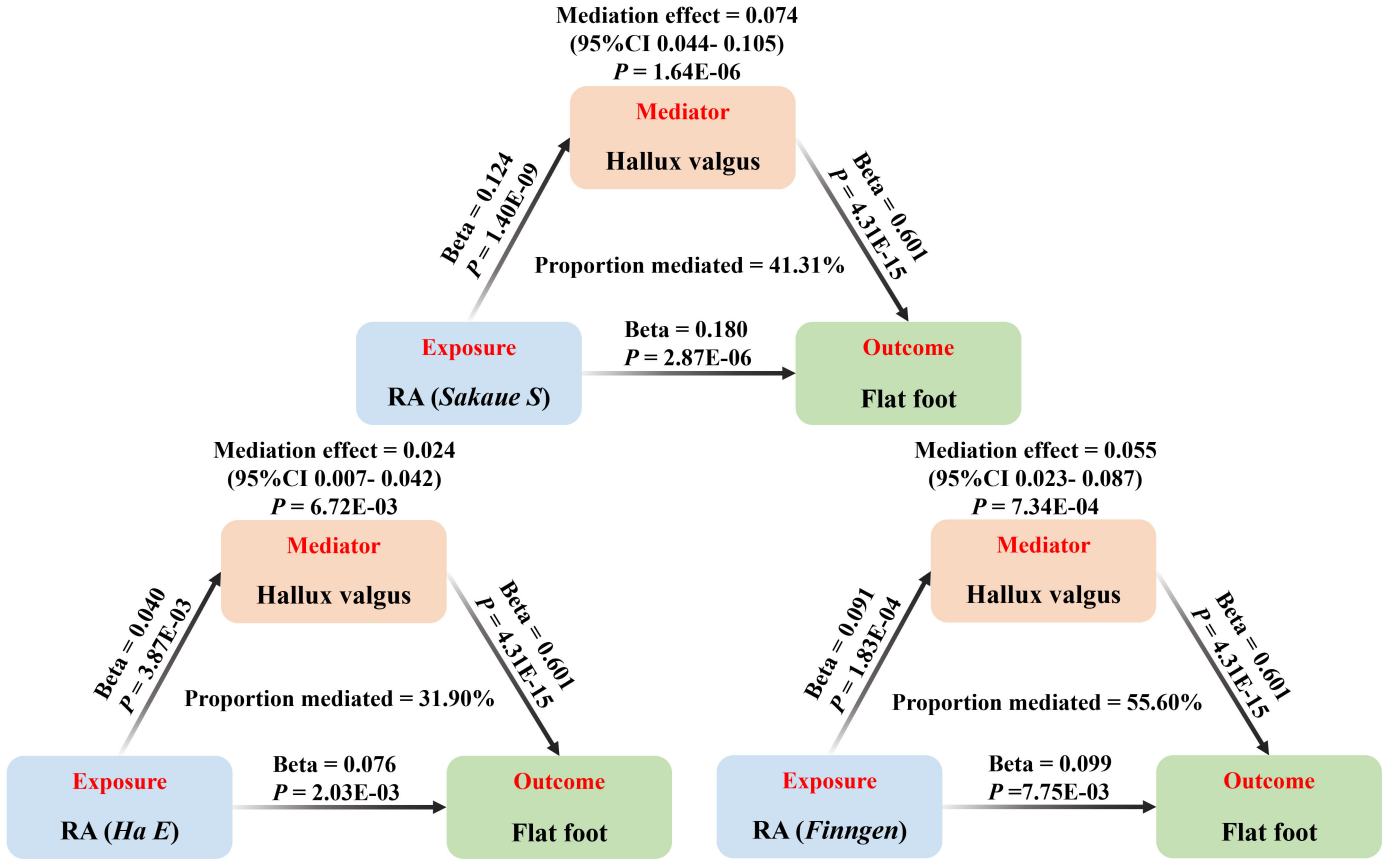
The mediation analysis of hallux valgus on the association between RA and flat foot (based on the IVW method), including the results of the main (Sakaue S) and replication (Ha E, Finngen) analyses.

The above results suggested hallux valgus may be a mediator of RA causing flat foot. The mediation effect of hallux valgus was calculated to be 0.074 (95% CI 0.044-0.105, *P* < 0.001) by two-step MR, with a mediation proportion of 41.31% (**Figure 4**; **Supplementary Table 9**). Replication analyses also validated the mediation effect of hallux valgus in two other European populations (**Figure 4**). Moreover, the mediation analysis based on the MRlap method showed the same direction as the main analysis, which indicated the mediation effect remained after controlling for the sample overlap rate (**Supplementary Figure 6**; **Supplementary Table 10**; **Figure 5**).

## 4 Discussion

This MR study investigated a potential causal relationship between RA and postural deformities, including flat foot, hallux valgus, and scoliosis. The results showed that genetically predicted RA was positively and causally associated with flat foot and hallux valgus but not significantly with scoliosis in a European population. In sensitivity analyses, Cochran’s Q test did not detect significant heterogeneity. The MR-Egger intercept test showed no significant horizontal pleiotropy. The cML-MA method and secondary analyses after obesity-associated SNP exclusion suggested the MR results were still robust with further control for horizontal pleiotropy. The directionality test confirmed the accuracy of the causality direction. Additionally, we analyzed the potential causal association among postural deformities. Surprisingly, a positive causality between hallux valgus and flat foot as well as scoliosis, suggesting hallux valgus may be a mediator of RA causing flat foot. Two-step MR confirmed the mediation effect of hallux valgus, and the results remained significant after controlling for the sample overlap rate by the MRlap method (**Supplementary Figure 2**).

Foot manifestations due to RA are initially usually in the forefoot and include hallux valgus, dorsal subluxation of the MTP joints, as well as hammer toe of the lesser toes, and get worse over time (36, 37). Previous observational studies have shown a positive association between RA and hallux valgus (38, 39). In a cross-sectional study in a Spanish population (220 RA patients with a median age of 59 years and a disease duration of 15.44 ± 10.54 years), hallux valgus was observed in 21.36% of the left foot and 22.28% of the right, respectively, in patients with RA progression of less than 10 years. Whereas with progression of more than 10 years, hallux valgus was observed in 30.92% of the left foot and 27.27% of the right (39). In another survey involving 955 Finnish RA patients (618 females and 337 males), the prevalence of hallux valgus was found to increase significantly with the duration of RA, and it was suggested rheumatoid inflammation of the medial sesamoid ligament may play an important role in the development of hallux valgus (40). Additionally, observational studies from non-European populations also found a correlation between RA and hallux valgus. Michelson et al. reported a 75.8% prevalence of hallux valgus, with moderate to severe hallux valgus in approximately 33% of cases, in an examination of 99 American patients with RA (age 58.4 ± 1.2 years, duration of disease 13.5 ± 1.1 years). Malhar H et al. examined 18 Indian RA patients (25 feet) not normally wearing shoes and found a 24% prevalence of hallux valgus present. However, UK RA patients (126 feet) who normally wore shoes had a 71.4% prevalence of hallux valgus (41).

Stolt et al. reviewed 32 studies published between 1981 and 2016 that reported a correlation between RA and foot problems, 21 of which originated from European populations (42). They found hallux valgus was the most common foot deformity problem in RA patients, affecting 35.0-65.3% of patients. Moreover, flat foot (11.0-42.1%) was also found to be more common in RA patients. The correlation between RA and flat foot was also reported in an observational study (154 RA cases and 101 controls) originating from France, which showed flat foot occurred significantly more frequently in RA patients than controls (*P* < 0.0005), especially in female patients (16). Results of another cross-sectional survey from Colombia showed that 42.1% of RA patients with a mean disease duration of 9.0 ± 7.1 years had flat foot (43). Additionally, a high correlation between RA and flat foot was reported in a study of Japanese (44).

Although RA often occurs in the small peripheral joints, it may still affect the spine. The incidence and factors associated with scoliosis, spondylolisthesis, and vertebral fracture in RA patients have been investigated in East Asian populations (17, 18). A Japanese prospective cohort study reported a 16% incidence of scoliosis in RA during a mean follow-up period of 7.0 ± 0.6 years, and multivariate analysis showed poor control of RA was an independent risk factor for *de novo* scoliosis (aOR 4.81, 95% CI 1.14-16.06, *P* = 0.011) (18). Another observational study from China found the prevalence of scoliosis to be 42.6% in RA patients, significantly higher than in controls (*P* = 0.002) (19). To our knowledge, there have been no reports on the correlation between RA and scoliosis in European populations. However, our results did not find a causal relationship between RA and scoliosis in European populations. Therefore, the results of MR studies originating in Europe may not be directly applicable to East Asian populations.

Hallux valgus and flat foot have been identified as co-features resulting from RA, but their association remains unclear. Bouysset et al. found a correlation between flat foot and metatarsus primus adductus in a study of the feet of 154 RA patients (mean age 55.6 years; mean disease duration 7.1 years) (16). Zafer et al. reported the calcaneal pitch angle was significantly lower in patients with hallux valgus than in normal patients (*P* < 0.001), and the number of patients with abnormal calcaneal pitch angle (*P* < 0.001) and talocalcaneal coverage angle (*P* = 0.03) was significantly higher in the case group than control, suggesting a high correlation between flat foot and hallux valgus (45). In contrast, Shi et al. reported no correlation between flat foot and splaying of forefoot in a study of the feet of 50 RA patients (disease duration >10 years) (46). Another study reported a new classification of hallux deformity (based on the horizontal and sagittal planes) applicable to RA patients and compared 5 clusters in relation to flat foot, finding that cluster III (boutonniere deformity type) correlated with flat foot but not cluster II (hallux valgus type) (44). Therefore, the relationship between hallux valgus and flat foot still requires further investigation, and our results provide evidence to support a causal relationship.

The correlation between RA and foot deformities may be mediated by several potential mechanisms. RA often results in peripheral joint erosive synovitis. The inflammatory process can lead to distension of the forefoot joint, triggering rupture of the collateral ligaments and plantar plates. Peripheral soft tissue destruction causes progressive joint instability in the foot in RA patients. Continuous motor loading may lead to dorsal subluxation of the metatarsophalangeal joints as well as plantar displacement of the metatarsal heads, leading to the development of hallux valgus. The development of flat foot may be related to the dysfunction of the posterior tibial tendon caused by RA. Patients with RA develop swelling and effusion around the posterior tibial tendon, and the inflammatory process leading to hindfoot joint destruction, subtalar joint instability, and tendon dysfunction may mediate the occurrence of flat foot and valgus deformity (47). Hallux valgus and flat foot are characteristic foot deformities in the progression of RA. Whether the first metatarsal bone adducts is due to the destruction of the surrounding ligaments by RA, which makes the support weaker and thus triggers the development of flat foot deformity remains controversial (16). However, our results support this mediating relationship.

The correlation between hallux valgus and scoliosis has been chiefly reported in dancers, but the causal relationship remains unclear (48, 49). The deformities may be related to extensive dance practice that exposes the spine and lower limb joints to high loads and strains. Scoliosis has been suggested to be a significant predictor of hallux valgus (OR = 2.09, 95% CI 1.11-3.92, *P* = 0.022). However, the results were derived from a cross-sectional study, which could not exclude confounding and define causality (49). In our study, the causal relationship between hallux valgus and scoliosis was determined by TSMR. Malalignment of the lower limb joints caused by this forefoot deformity may distort the directions of forces and pass across the joints, which in turn triggers the development of undesirable spinal deformities such as scoliosis (50). Consequently, our results could contribute to better standardization of their training programs as well as screening strategies to prolong careers.

Despite numerous studies reporting the correlation between RA and postural deformities, as well as among postural deformities, these observational studies cannot clarify the causality between the diseases, which usually requires extensive cohort studies to confirm (51). To our knowledge, this has not been reported. The MR provides a more efficient, cost-effective, and highly feasible approach (26). We used MR to clarify for the first time the causal relationship between RA and hallux valgus and flat foot, and we also identified hallux valgus as a mediator of RA causing flat foot; the causality between hallux valgus and scoliosis was also confirmed. This study has significant clinical implications for the management of RA and associated postural deformities. First, the identification of hallux valgus as a mediator between RA and flat foot suggests that early detection of hallux valgus could serve as a valuable predictive marker, enabling preventive interventions before severe deformities develop. This underscores the need for comprehensive foot assessments in RA patients, regardless of disease severity. Customized orthotics, modified footwear, and targeted physiotherapy could enhance mobility and function, ultimately improving the quality of life for patients with rheumatoid foot problems (52). Moreover, aggressive pharmacological treatment of RA, particularly with DMARDs or traditional Chinese medicine formulations, could play a key role in preventing foot deformities by controlling inflammation and joint destruction, potentially reducing the need for surgical correction. However, despite these opportunities for prevention, studies show that only one-third of RA patients receive any form of foot intervention, highlighting a gap in current care (53). Raising awareness among healthcare providers and ensuring that orthopedic surgeons with expertise in foot deformities are actively involved in multidisciplinary teams along with rheumatologists and other specialists can significantly improve outcomes.

In this study, two replication analyses were performed to strengthen the results of the primary analysis of causality between RA and postural deformities. Although horizontal pleiotropy was not detected by the MR-Egger intercept test, we validated it by using the cML-MA method and secondary analysis after confounders exclusion, which controlled for horizontal pleiotropy to a greater extent. Since the data sources of the three postural deformities were all from the Finngen database, there may be a possibility of biased results caused by the sample overlap rate when performing MR among them. Although some studies have reported that two samples from a single large database can be safely analyzed by MR, we still used the MRlap method to correct the analysis for the group with a large sample overlap rate and obtained results consistent with previous (54). There were also some limitations in this study: due to the limited source of data related to postural deformities, only from European populations, the results may not be generalizable to other populations, which would require additional analyses of other ethnicities at a later stage.

## 5 Conclusion

In conclusion, the results of our MR study demonstrate the causal and mediating relationships between RA and postural deformities in a European population species. This study may provide new insights into the prevention of foot deformities in RA patients. Early screening and treatment of RA patients and wearing orthopedic devices may reduce the incidence of foot deformities and improve patients’ quality of life.

## Data Availability

The original contributions presented in the study are included in the article/**Supplementary Material.** Further inquiries can be directed to the corresponding authors.
