# Supplementary material for: The causality between rheumatoid arthritis and postural deformities: bidirectional Mendelian randomization study and mediation analysis
Source: Front Immunol. 2024 Oct 3;15:1453685. doi: 10.3389/fimmu.2024.1453685 (PMC11484279; doi:10.3389/fimmu.2024.1453685)
Supplement: Supplementary file 1 [file DataSheet1.docx]

**The causality between rheumatoid arthritis and postural deformities: bidirectional Mendelian randomization study and mediation analysis**

Piqian Zhao, Zhe Chen, Ya Wen, Hongtao Zhang, Liangyuan Wen, Zijie Pei.

| **Table of Contents** | **Numbered pages** |
| --- | --- |
| Supplementary Figure 1. Genetic correlations LDSC analysis | 1 |
| Supplementary Figure 2. Scatter plot for MR analysis of the causal association between RA and postural deformities | 1 |
| Supplementary Figure 3. Funnel plot for MR analysis of the causal association between RA and postural deformities | 2 |
| Supplementary Figure 4. MR leave-one-out sensitivity analysis of the causal association between RA and postural deformities | 3 |
| Supplementary Figure 5. Scatter plot, funnel plot and MR leave-one-out sensitivity analysis of the causal association between hallux valgus and both scoliosis and flat foot | 4 |
| Supplementary Figure 6. Path diagram for the mediation analysis of hallux valgus on the association between RA and flat foot (based on the results of the MRlap method) | 4 |
| Supplementary Table 1. STROBE-MR checklist | Supplementary Table |
| Supplementary Table 2. Instrumental variables | Supplementary Table |
| Supplementary Table 3. Genetic correlations evaluated by LDSC regression analysis | Supplementary Table |
| Supplementary Table 4. MR analysis results between RA and postural deformities | Supplementary Table |
| Supplementary Table 5. MR analysis results between RA and both hallux valgus and flat foot (removing obesity-related SNPs) | Supplementary Table |
| Supplementary Table 6. Reverse MR analysis results | Supplementary Table |
| Supplementary Table 7. MR analysis results between postural deformities | Supplementary Table |
| Supplementary Table 8. MR analysis results based on MRlap method | Supplementary Table |
| Supplementary Table 9. Mediation analysis results based on the results of the IVW method | Supplementary Table |
| Supplementary Table 10. Mediation analysis results based on the results of the MRlap method | Supplementary Table |


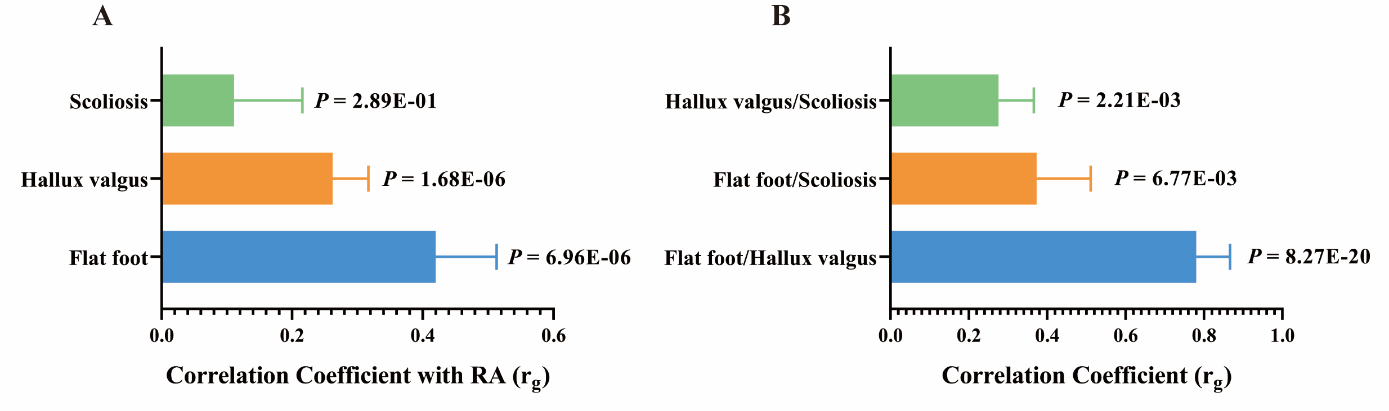


**Supplementary Figure 1.** Genetic correlations evaluated by LDSC regression analysis. **A** The genetic correlations between RA and postural deformities. **B.** The genetic correlations between postural deformities.


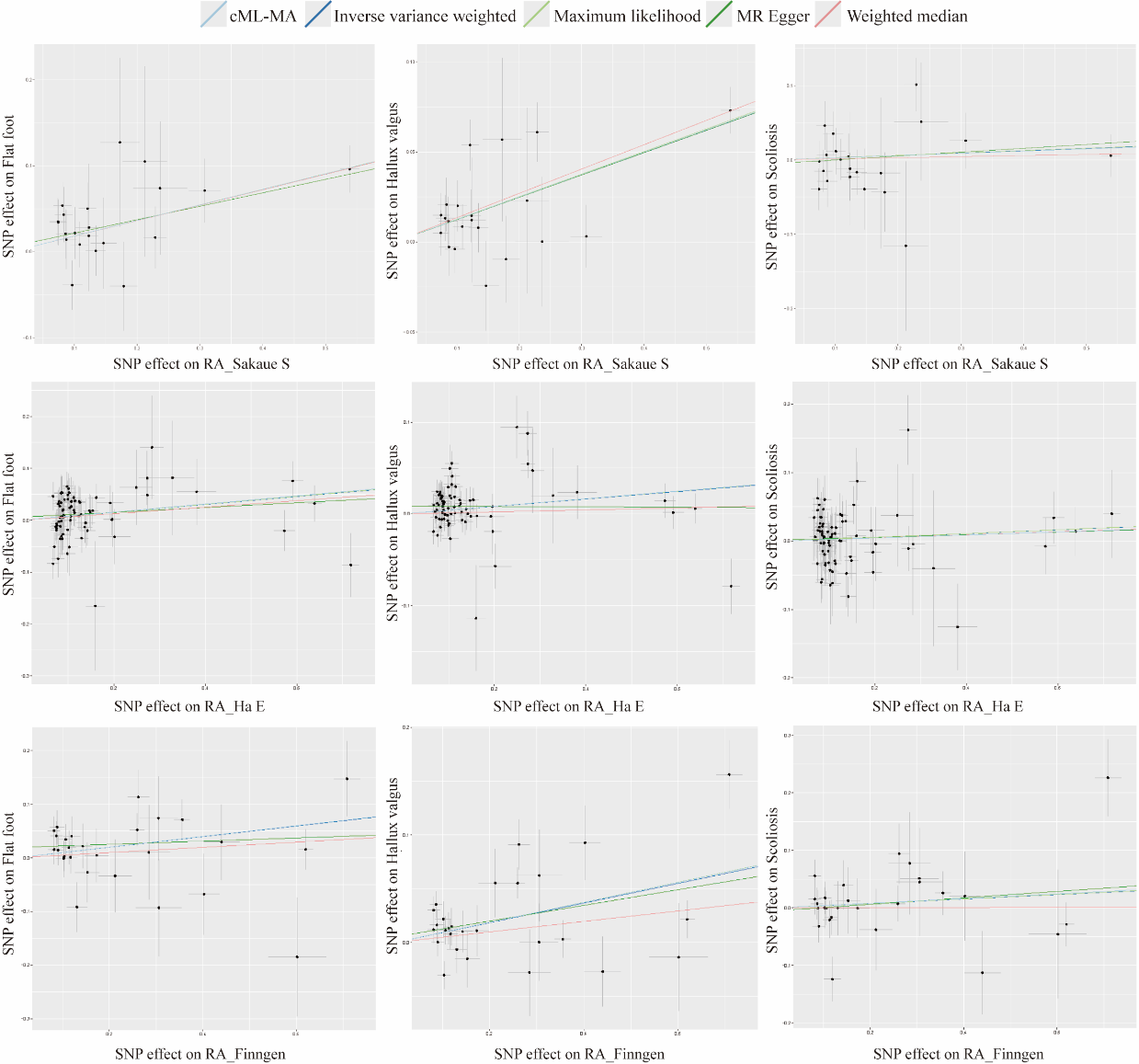


**Supplementary Figure 2** Scatter plot for MR analysis of the causal association between RA and postural deformities, including the results of the main (Sakaue S) and replication (Ha E, Finngen) analyses. The lines represent the visualization of five MR analysis results, with slope showing the effect size.


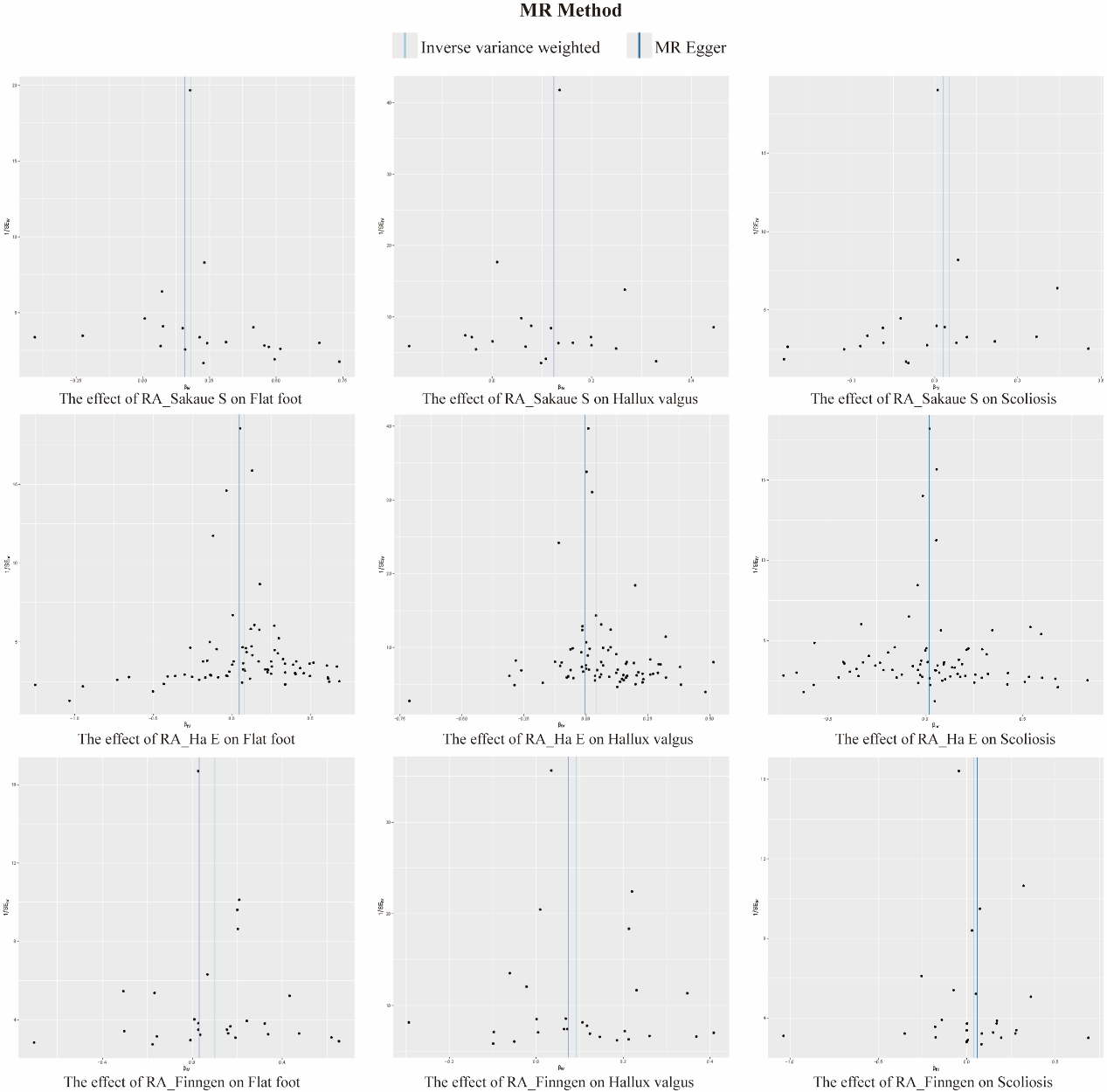


**Supplementary Figure 3** Funnel plot for MR analysis of the causal association between RA and postural deformities, including the results of the main (Sakaue S) and replication (Ha E, Finngen) analyses.


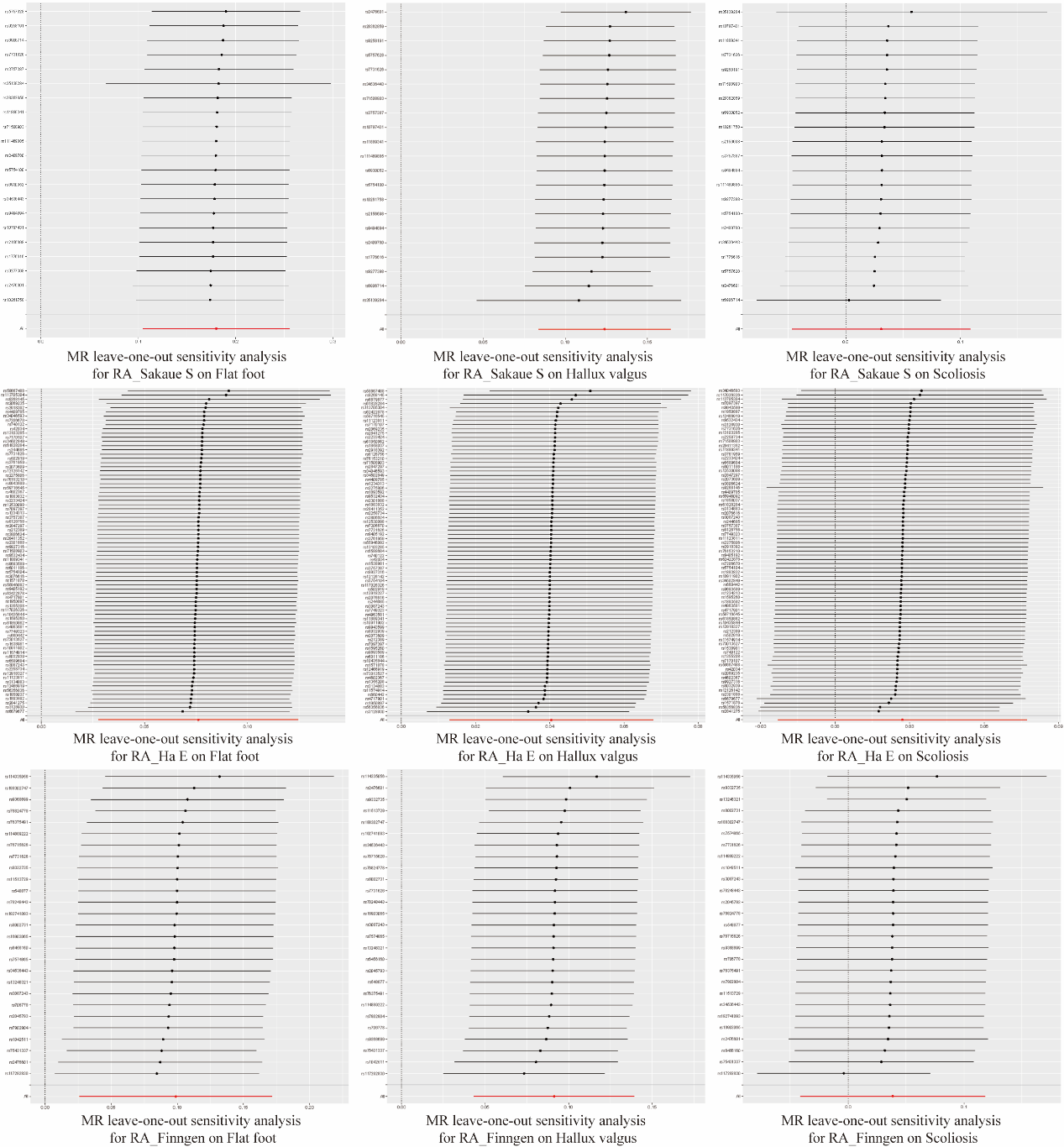


**Supplementary Figure 4** MR leave-one-out sensitivity analysis of the causal association between RA and postural deformities, including the results of the main (Sakaue S) and replication (Ha E, Finngen) analyses.


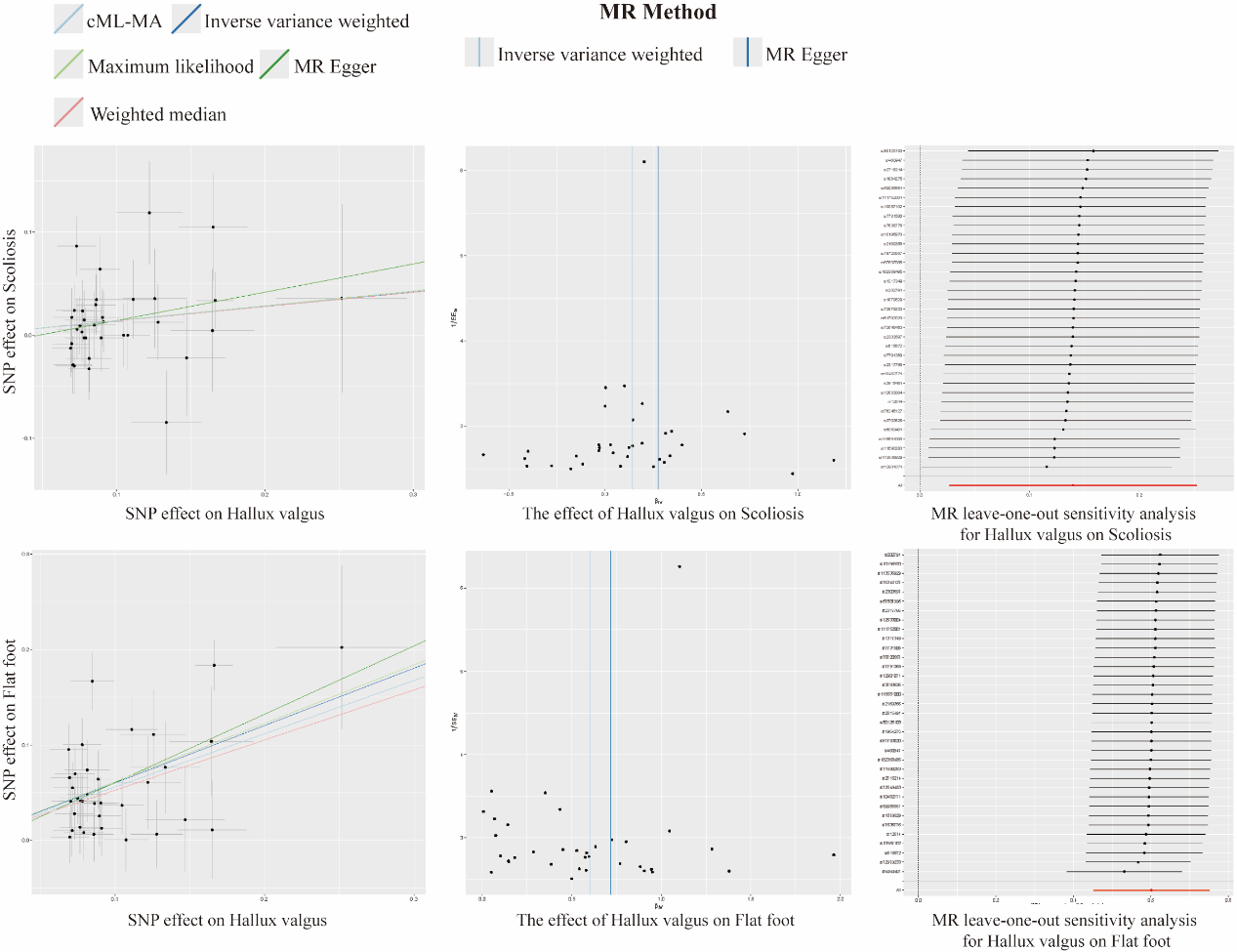


**Supplementary Figure 5** Scatter plot, funnel plot and MR leave-one-out sensitivity analysis of the causal association between hallux valgus and both scoliosis and flat foot.


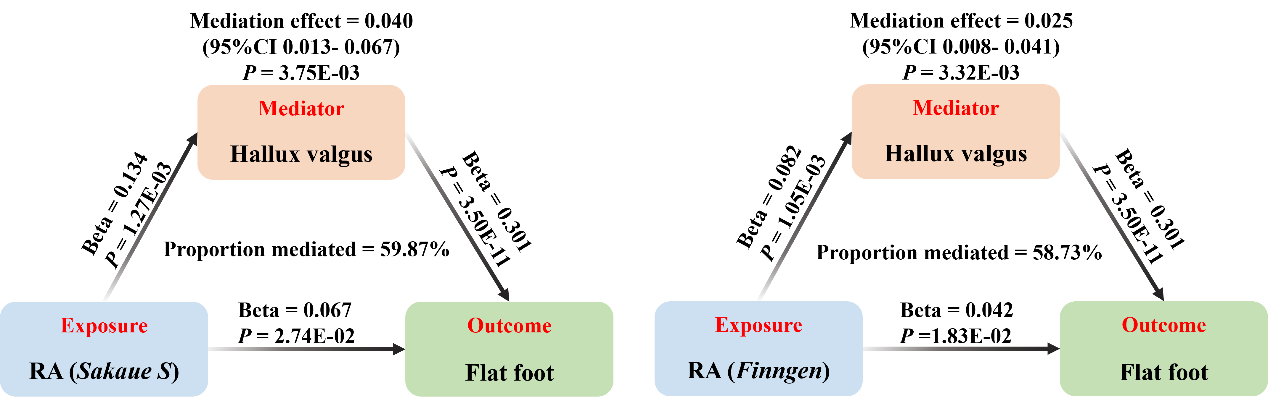


**Supplementary Figure 6** Path diagram for the mediation analysis of hallux valgus on the association between RA and flat foot (based on the results of the MRlap method), including the results of the main (Sakaue S) and replication (Finngen) analysis.
